# Quantification of Lung Fibrosis and Emphysema in Mice Using Automated Micro-Computed Tomography

**DOI:** 10.1371/journal.pone.0043123

**Published:** 2012-08-13

**Authors:** Ellen De Langhe, Greetje Vande Velde, Jeroen Hostens, Uwe Himmelreich, Benoit Nemery, Frank P. Luyten, Jeroen Vanoirbeek, Rik J. Lories

**Affiliations:** 1 Laboratory for Skeletal Development and Joint Disorders, Department of Development and Regeneration, KU Leuven, Leuven, Belgium; 2 Department of Rheumatology, University Hospitals Leuven, Leuven, Belgium; 3 Biomedical NMR Unit/MoSAIC, Department of Imaging and Pathology, KU Leuven, Leuven, Belgium; 4 SkyScan, Kontich, Belgium; 5 Research Unit of Lung Toxicology, Department of Public Health, KU Leuven, Leuven, Belgium; McMaster University, Canada

## Abstract

**Background:**

*In vivo* high-resolution micro-computed tomography allows for longitudinal image-based measurements in animal models of lung disease. The combination of repetitive high resolution imaging with fully automated quantitative image analysis in mouse models of lung fibrosis lung benefits preclinical research. This study aimed to develop and validate such an automated micro-computed tomography analysis algorithm for quantification of aerated lung volume in mice; an indicator of pulmonary fibrosis and emphysema severity.

**Methodology:**

Mice received an intratracheal instillation of bleomycin (n = 8), elastase (0.25U elastase n = 9, 0.5U elastase n = 8) or saline control (n = 6 for fibrosis, n = 5 for emphysema). A subset of mice was scanned without intervention, to evaluate potential radiation-induced toxicity (n = 4). Some bleomycin-instilled mice were treated with imatinib for proof of concept (n = 8). Mice were scanned weekly, until four weeks after induction, when they underwent pulmonary function testing, lung histology and collagen quantification. Aerated lung volumes were calculated with our automated algorithm.

**Principal Findings:**

Our automated image-based aerated lung volume quantification method is reproducible with low intra-subject variability. Bleomycin-treated mice had significantly lower scan-derived aerated lung volumes, compared to controls. Aerated lung volume correlated with the histopathological fibrosis score and total lung collagen content. Inversely, a dose-dependent increase in lung volume was observed in elastase-treated mice. Serial scanning of individual mice is feasible and visualized dynamic disease progression. No radiation-induced toxicity was observed. Three-dimensional images provided critical topographical information.

**Conclusions:**

We report on a high resolution *in vivo* micro-computed tomography image analysis algorithm that runs fully automated and allows quantification of aerated lung volume in mice. This method is reproducible with low inherent measurement variability. We show that it is a reliable quantitative tool to investigate experimental lung fibrosis and emphysema in mice. Its non-invasive nature has the unique benefit to allow dynamic 4D evaluation of disease processes and therapeutic interventions.

## Introduction

Pulmonary fibrosis, either idiopathic or secondary to diseases such as systemic sclerosis, is a devastating and life-threatening condition, for which effective treatments are still lacking [1]. Rodent animal models are commonly used to unravel fibrotic processes in the lung and to develop new therapeutic strategies. Different mouse models of fibrosis are available and include bleomycin-induced lung fibrosis, irradiation-induced fibrosis, lung-specific transgenic mice, and adenoviral vector-delivered gene overexpression models. However, no current animal model recapitulates all features of the human disease [2]. The bleomycin-induced pulmonary fibrosis model is well characterized and results in rapid, dose-dependent fibrosis induction. Although its direct clinical relevance as a model for human idiopathic fibrosis is debated, it is the most extensively used rodent model [3]. Intratracheal administration results in direct epithelial damage with prominent pan-alveolitis within the first week. Transient fibrosis develops from day 14 onwards, with maximal responses around days 21 to 28 and spontaneous resolution afterwards. Evaluation of disease severity in these models is primarily based on end-stage procedures including histopathology and collagen, hence precluding dynamic evaluation of disease progression in individual mice.

*In vivo* imaging of mouse lungs with micro-computed tomography (µCT) has been incorporated into preclinical research, but remains technically challenging due to respiratory movement artifacts [4]–[7]. Rapid, non-invasive, serial imaging of animal models should ideally result in quantitative datasets that allow for longitudinal assessment, comparisons between different groups including the effect of therapeutic interventions, and detailed topographic information documenting the extent of disease in the individual animal. µCT-based protocols for the quantification of pulmonary fibrosis in mice have been proposed but face further challenges in combining high spatial resolution, longitudinal setup, automation of the analysis and agreement with standards of histopathological or biochemical analysis [6], [8]–[10]. Moreover, these protocols are generally labor intensive and require specific radiological skills [6], [11].

Here, we report on a fully automated algorithm for the longitudinal *in vivo* quantitative assessment of pulmonary fibrosis in mice using high-resolution µCT. We propose this imaging technique as a reliable and quantitative tool that yields dynamic information on disease processes. Our imaging and analysis protocol permits comparison between different groups, enables evaluation over time in individual animals and provides specific topographic information on the processes of fibrosis. Moreover, we confirm the validity and thereby extend the applicability of our lung volume analysis algorithm in the elastase-induced mouse model of pulmonary emphysema. This model is known to induce severe dose-dependent emphysematous disease with rapid onset after a single intratracheal instillation, a preferred model to study airspace enlargement, albeit with lesser direct clinical relevance compared to the long-term cigarette smoke-induced mouse model of emphysema [12].

## Materials and Methods

### Ethics statement

The KU Leuven Ethical Committee for animal research approved all experiments. Institutional guidelines for animal welfare and experimental conduct were followed.

### Animal models

Eight-week old male C57Bl/6 mice (Janvier, Le Genest, France), weighing 22–25 grams, were used. Mice were anaesthetized for the different procedures as indicated below. The reproducibility of the technique was evaluated in a separate experiment by analyzing data from 4–5 consecutive scans from individual mice. Fibrosis was induced by intratracheal instillation of 0.05U bleomycin (Sanofi-Aventis, Diegem**,** Belgium) in 50 µl of sterile PBS or PBS as a control, after intra-peritoneal anesthesia with ketamine and xylazine. A group of mice was treated with imatinib (Novartis, Vilvoorde, Belgium) (50 mg/kg/d i.p.). Imatinib was purchased from our hospital pharmacy in 100 mg capsules and was processed as previously described [13], dissolved in distilled H_2_O to a final concentration of 12.5 mg/ml, aliquoted and stored at −20° until usage. Pulmonary emphysema was induced by intratracheal instillation of 0.25U or 0.5U of porcine pancreatic elastase (PPE) in 50 µl of sterile PBS. To evaluate possible radiation-induced toxicity, a subset of mice was scanned weekly, without other intervention. The number of mice used in the different experiments is shown in Table 1.

**Table 1:** Number of mice used in the experiments.

Type of experiment	Intervention	Baseline (n)	End-stage (n)^1^
Scan toxicity	None^2^	4	4
Repeatability	Bleomycin	5	3
Pulmonary fibrosis feasibility	PBS	6	6
	Bleomycin	8	6
Pulmonary fibrosis validation	PBS	5	5
	Bleomycin	6	6
Pulmonary fibrosis therapy	Bleomycin + Imatinib	8	6^3^
Pulmonary emphysema	PBS	5	5
	0.25U PPE	9	9
	0.5U PPE	8	8

1 Number of mice at end-stage represents survivors in the experiment^ 2^ Mice in this analysis were scanned 4 times without any other intervention. ^3^ Only 4 mice had successful scans at all time points and were included in the longitudinal analysis.

### µCT imaging

Mice were scanned in supine position using a desktop *in vivo* small animal μCT (SkyScan 1076, software version 3.2, Kontich, Belgium) at day 7, 14, 21 and 28 after induction. Mice were sedated with 2% isoflurane/2l O2 flow inhalation anesthesia by a nose cone (Figure S1). During image acquisition thoracic breathing movements were recorded with a visual camera, detecting the up- en downward movement of the thorax. This was translated into a pseudo-sinusoidal signal to allow retrospective respiratory gating. The length of a complete respiratory cycle was divided into 4 phases of identical length, corresponding to 4 phases of the respiratory cycle, from the initiation of inspiration to end-expiration. Images were acquired throughout the spontaneous respiratory cycle. Post-acquisition, all images were sorted in the corresponding phase of respiration in which they were acquired, thereby significantly reducing movement artifacts caused by respiration. The number of groups (4: bin 0 to 3) was optimally set for our image acquisition settings, resulting in at least 2 projection images per bin. For Hounsfield Units (HU) calibration, a phantom (air-filled 1.5 ml tube, inside of a water-filled 50 ml tube) was scanned. Based on full stack histograms of a manually delimited volume-of-interest, containing only water or air, the mean grayscale index of water (81.32) was set at 0 HU, and grayscale index of air (0) at −1000 HU. Images were acquired in list-mode with the following parameters: 50 kVp X-ray source voltage, 180 µA current, a composite X-ray filter of 0.5 mm aluminium, 120 msec camera exposure time per projection, 9 projections per view, 23×35 mm field of view, acquiring projections with 0.7° increments over a total angle of 180°, producing images with a real pixel size of 35 µm. Total scanning time was approximately 12 minutes, resulting in a radiation dose of 813 mGy. Tomograms were reconstructed using NRecon software (version 1.6.1.3, SkyScan). Reconstruction parameters were smoothing “2”, beam-hardening correction “31%”; post-alignment and ring artifact correction were optimally set for each individual scan. Reconstructed 3D datasets had an isotropic 35 µm voxel size and 1000×1000 resolution.

Following pulmonary function tests (see below), tracheotomized mice were euthanized by pentobarbital overdose and scanned for pressure-volume curve construction. After euthanasia, tracheotomized mice were transferred again to the µCT after which decreasing pressures (30-3 cmH_2_O) were applied to the lungs via the tracheal cannula and lungs were scanned for pressure-volume curve construction. For this purpose, image acquisition parameters were 55 kVp X-ray source voltage, 180 µA current, a composite X-ray filter of 0.5 mm aluminium, 120 msec camera exposure time per projection, 23×35 mm field of view, acquiring projections with 0.7° increments over a total angle of 180°, averaging 3 frames, producing images with a real pixel size of 35 µm. Total scanning time was approximately 5 minutes per pressure point. Images were reconstructed with identical settings as previously described.

### Pulmonary function tests

Freely breathing, isoflurane-sedated mice were scanned 28 days after induction, as described above. Subsequently, deeper anesthesia was achieved with an i.p. injection of pentobarbital (70 mg/kg) (CEVA, Diegem, Belgium) to suppress spontaneous breathing. After a tracheostomy, the mice were connected to the flexiVent system (SCIREQ, Montreal, Canada) [14]. The computer-controlled small animal ventilator ventilated the mice quasi-sinusoidally with a tidal volume of 10 ml/kg at a frequency of 150 breaths/minute and a positive end-expiratory pressure of 2 cmH_2_O to achieve a mean lung volume close to that during spontaneous breathing. On the flexiVent we performed two perturbations: A snapshot perturbation and a pressure-volume loop with constant increasing pressure (PVr-P). Each time before performing these perturbations, a total lung capacity perturbation (TLC) was carried out to normalize the lungs. The snapshot and PVr-P perturbations were performed until three acceptable measurements (coefficient of determination = COD>0.95) were recorded in each individual subject, of which an average was calculated. The snapshot perturbation was imposed to measure resistance, compliance, and elastance of the whole respiratory system (airways, lung, and chest wall). The PVr-P perturbation generated vital (total) lung capacity (A), inspiratory capacity from zero pressure (B), form of deflating PV loop (K), static compliance (Cst) and static elastance (Est) and hysteresis (area between inflating and deflating part of the PV loop). Raw data from the PVr-P perturbation was used to reconstruct PV-curves. The presented PV-loops are group averages (n = 4–6 per group).

### Image analysis

Two different algorithms were developed for segmentation of aerated lung volumes in the euthanized and *in vivo* settings (CTAn software, version 1.10.0.0, SkyScan). For analysis of images from euthanized animals, acquired images were first modified by a thresholding procedure, excluding all pixels with densities > −580 HU. This threshold was based on the reconstructed images of a phantom with an air-containing Eppendorf tube. The complete histogram of air was situated at < −580 HU. This first thresholding step results in automatic segmentation of pixels with a density corresponding to pure air. This results in selection of air within the lungs but also includes all air surrounding the animal, since the region of interest (ROI) still equals the total field of view. When applying a ‘despeckle’ maneuver, removing all air-containing pores in 2D, only the air outside the animal will be segmented. When equating the ROI to a copy of the image, this results in an ROI that only contains the animal. When the image is then reloaded and is equated to the intersection of the ROI and the image, it results in an ROI that equals the image of the animal. From this image, full stack histograms are calculated in grayscale indices and all data from −1000 to +1000 HU were plotted. This ROI is next adapted using a morphological operation that erodes 10 pixels at the border of the ROI to sharpen the resulting shape. When the image is reloaded and thresholding is repeated (excluding all pixels > −580 HU), this results in segmentation of all areas of air within the animal. This segmentation is still polluted by pixels outside the lung with low densities (e.g. in subcutaneous fat) which can be eliminated by repeating a despeckle maneuver (sweep), which will eliminate all but the largest object. At this point, the algorithm results in the specific segmentation of aerated lung volume. In the next step, 3D parameters are calculated, resulting in volume data in voxel number and mm^3^ and allowing 3D image construction using CTVol software (SkyScan, version 1.11.1.2). This analysis algorithm was run on all available datasets.

In freely breathing animals, retrospective respiratory gating minimized, but did not eliminate the unsharp borders of moving organs (e.g. heart, lungs). The algorithm was therefore adapted to correct for intrinsic pixel contamination at those borders by using a higher threshold for air segmentation. Upon visual inspection of reconstructed images in Dataviewer (SkyScan) of lung regions directly above the diaphragm in inspiration and expiration, the discriminating threshold for air was set at < −383 HU to capture the inflation of lungs upon inspiration. This was performed only once, at the start of the experiment and this density threshold was used automatically for all consecutive analyses. Total segmentation time is 10 minutes per scan and required no interference from the investigator. Since datasets corresponding to 4 different phases of the breathing cycle are available, 4D evaluation of the air in the lung is feasible. End inspiratory volume (EIV) was defined as the calculated volume at the end of inspiration, and was found in bin 0. End expiratory volume (EEV) (corresponding to Functional residual volume (FRV)) was defined as the calculated volume at the end of expiration, and was found in bin 3. The EEV was used in all analyses as it evaluates air trapping and is not influenced by anesthesia-induced gasping.

### Histological analysis

After euthanasia and completion of µCT imaging, the tracheal cannula was removed, the chest cavity was opened and heart and lungs were removed en bloc. The left lung was collected for histopathology, inflated with 400 µl of 10% formalin/PBS via the left main bronchus and fixed in formalin for 24 hours. After paraffin embedding, 5 µm sections were cut throughout the whole lung. Five sections, with 1 mm interval, were stained with hematoxylin-eosin. The validated semiquantitative Ashcroft score was used to score pulmonary fibrosis [15]. In short, upon 100× magnification, each successive field was given a score ranging from 0 (normal lung) to 8 (total fibrous obliteration of the field). All scores from 5 sections were averaged.

### Hydroxyproline quantification

The right lung lobes were collected for hydroxyproline quantification and stored at −80°C for later analysis. Hydroxyproline quantification was performed as previously described [16]. In short, right lung lobes were hydrolyzed for 3 hours in 6M HCl at 120°. After cooling down for 15 minutes, pH was neutralized (pH 6–7) using 500 µl NaOH. Samples were diluted 1/20 in sterile H_2_0. Free hydroxyproline was oxidized with Chloramine-T for 20 minutes after which the oxidation reaction was stopped using 70% PCA. Ehrlich's reagent was added and samples were heated for 20 minutes in a 60° water bath. After cooling down for 5 minutes, absorbance was measured at 570 nm and concentrations were calculated based on a standard curve.

### Statistics

Data were analyzed using GraphPad Prism 5.00 (GraphPad Software Inc., San Diego, CA). Normal distribution was assessed visually by plots and with the Kolmogorov-Smirnov test. Repeatability (measurement of error) was determined as proposed by Bland and Altman [17]. Intra-subject variance and standard deviation were determined for repeated measurements on 5 normal and 3 fibrotic mice (n = 4 or 5 measurements per mouse). Equal variances were checked by plotting mean vs. standard deviation and by the Spearman correlation. Within-subject standard deviation (sw) was determined by one-way ANOVA as the square root of the residual mean square value. 95% repeatability coefficients were determined as 1.96 * √2 * sw and represent the range of repeated measurements. The agreement between µCT derived volume calculations and standard measurement was analyzed by linear regression and visualized using 95% prediction lines [17].

Differences between two groups were analyzed by t-tests with Welch correction for unequal variances if necessary. Post-hoc and a priori power calculations were performed G*Power software (version 3.1.3 for Mac) using double-sided 5% alpha error and a minimum power of 80%. An overview of the power calculations is given in Table 2. One-way ANOVA with Newman Keuls *post-hoc* tests was used for multiple group comparisons. Two-way ANOVA was used for the analysis of repeated measurements in longitudinal analysis experiments. P-values <0.05 were considered significant.

**Table 2:** post hoc and a priori power analyses of the different experiments.

	Analysis	Effect size	Power	Sample size
**Pulmonary fibrosis: bleomycin vs. control**				
µCT^1^	Post hoc	1.861	78%	11 (5 vs 6)
	A priori	1.861	80%	12 (6 vs 6)
			95%	18 (9 vs 9)
Ashcroft^1^	Post hoc	4.675	99%	10 (4 vs 6)
Hydroxyproline^1^	Post hoc	6.595	100%	8 (4 vs 4)
Area under curve Pressure-Volume^1^	Post hoc	2.167	88%	11 (5 vs 6)
	A priori	2.167	80%	10 (5 vs 5)
			95%	14 (7 vs 7)
**Pulmonary fibrosis: bleomycin vs. imatinib**				
µCT endstage^1^	A priori	0.93	80%	36 (19 vs 19)
µCT timecourse (bleomycin vs bleomycin + imatinib)^2^	Post hoc	0.761	71%	9 (4 vs 5)
	A priori	0.761	80%	10 (5 vs 5)
			95%	16 (8 vs 8)
µCT timecourse (bleomycin vs bleomycin + imatinib + control)^2^	Post hoc	0.808	91%	16 (5 vs 5 vs 7)
	A priori	0.808	80%	15 (5 vs 5 vs 5)
			95%	21 (7 vs 7 vs 7)
**Pulmonary emphysema**				
µCT^3^	Post hoc	1.441	99%	22 (5 vs 9 vs 8)
	A priori	1.441	80%	9 (3 vs 3 vs 3)
			95%	12 (4 vs 4 vs 4)

1 analyzed for unpaired t-test, ^2^ Two way ANOVA for repeated measurements, ^3^ One way ANOVA (3 groups).

## Results

### Reproducible quantitative and qualitative assessment of aerated lung volumes by in vivo µCT imaging

**Figure 1:**
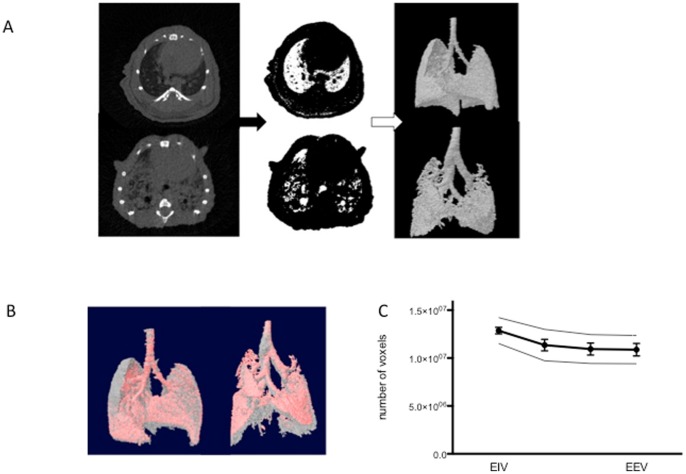
Qualitative and quantitative assessment of lung volume by *in vivo* µCT imaging. (A) The crucial steps of the automated analysis protocol are illustrated in a normal and a fibrotic lung. On each tomogram, pixels with grayscale indices below 50 Houndsfield Units (HU) are selected, segmenting air-containing pixels. Further despeckling and thresholding steps eliminate contamination by extrapulmonary air and result in stack tomogram-based 3D parameter calculation and reconstruction of a 3D model that visualizes aerated lung volumes. Note that the mouse has four lobes in the right lung with one retrocardiac lobe. (B) Retrospective gating allowing surface rendering of inspiratory (gray) and expiratory (red) air volumes. (C) Calculated aerated lung volumes of a representative normal lung, scanned 4 consecutive times in 1 day, expressed in total voxel number, plotted versus respiratory cycle phase divided into four phases and extending from the end of inspiration (EIV) to the end of expiration (EEV). (Data are mean +/- SD, additional thin lines above and below the main line are mean + and – repeatability coefficients, respectively (n = 5 mice)).

Mouse lungs were scanned *in vivo* using inhalation anesthesia and simultaneous video-registration of the breathing movements. All high-resolution tomograms obtained *in vivo* were progressively segmented to separate the air-containing lung spaces from the rest of the image. In the bleomycin-induced model, the heterogeneous loss of aerated lung volume is clearly distinguishable from the normal lung and reflects restrictive lung disease (Figure 1A). Reconstructed 3D images allowed visualization of the topographical distribution of fibrosis. The applied algorithm calculated 3D volumes in four automatically defined stages of the respiratory cycle using retrospective gating based on the simultaneous video recording. Merging of the 3D images obtained at the extremes resulted in an intuitive 4D data representation (Figure 1B). In the fibrotic lung, recruitment of air space upon inhalation was restricted as compared to control lungs. In addition to the qualitative visual data, calculated 3D parameters of the *in vivo* µCT images resulted in a quantitative volume output, expressed as total voxel number, per phase of the breathing cycle, from end-inspiratory volume (EIV) to end-expiratory volume (EEV) (Figure 1C).

Repeatability or measurement error of this quantitative volume assessment was checked for normal and fibrotic lungs (n = 5 and 3 mice respectively) by analyzing data from 4 or 5 identical and consecutive scans per individual mouse. Variance of the volumes was not correlated with magnitude (Spearmann correlation, r =  −0,09, p = 0.8). The within subject standard deviation (sw) was 4,8*10^-5^ and 5,3*10^-5^ voxels for EIV and EEV respectively, resulting in repeatability coefficients of 1,35*10^-6^ and 1,47*10^−6^ voxels. This value indicates that 95% of paired readings on the same mouse will lie within these limits and suggests low measurement error (Figure 1C).

### Validation of µCT imaging in murine lung fibrosis

The potential value of the µCT imaging technique as an outcome measure in preclinical studies of lung fibrosis is defined by its agreement with established standards such as histomorphological analysis and lung collagen content quantification. In this context µCT imaging could become an excellent tool with the promise to quantitatively evaluate fibrosis severity without the need to perform end-stage procedures, also allowing longitudinal follow-up of an individual animal.

**Figure 2:**
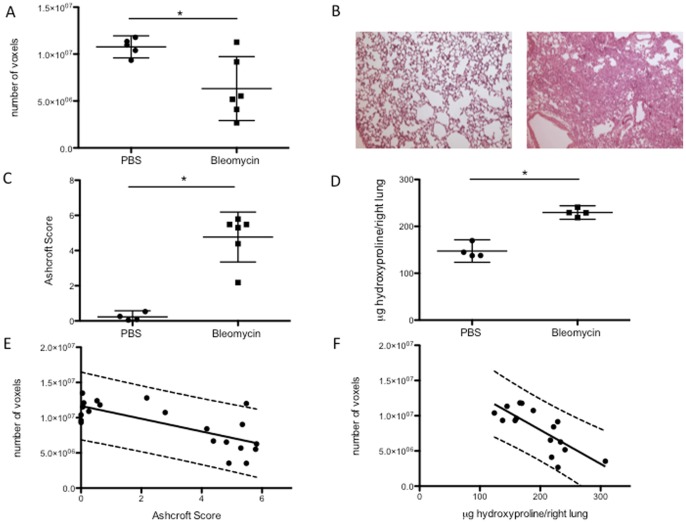
Validation of µCT-based quantification of aerated lung volumes in fibrosis. (A) End-expiratory aerated volumes (EEV), calculated by µCT, in bleomycin-induced pulmonary fibrosis (data are mean and 95% CI, * p = 0.024). (B) Representative histology of a saline (PBS) - (left) and a bleomycin- (right) treated mouse (hematoxylin & eosin staining). (C) Ashcroft score (data are mean and 95% CI, * p = 0.0005). (D) Total collagen content (data are mean and 95% CI, * p = 0.0007). (E) Agreement between EEV and histopathological fibrosis (F) and hydroxyproline content (G) based on linear regression (R^2^  = 0.523; p = 0.0002 and R^2^  = 0.598; p = 0.0004 respectively). Plots show the linear regression line and 95% prediction intervals.

Four weeks after bleomycin instillation aerated EEV calculated by µCT analysis showed a significant reduction of lung volume by bleomycin treatment (p = 0.024 – n = 5 vs 6). (Figure 2A). Pulmonary fibrosis was confirmed by histology with extensive deposition of extracellular matrix at day 28 after bleomycin instillation (Figure 2B). Ashcroft histopathology score was significantly increased in bleomycin-treated mice (p = 0.0005 – n = 4 vs 6) (Figure 2C). Collagen content of the lungs quantified by hydroxyproline determination confirmed the successful induction of fibrosis by bleomycin (p = 0.0007 – n = 4 vs 4) (Figure 2D). Repetitive scanning only did not alter Ashcroft score or total lung collagen content as compared to normal lung (data not shown). This first dataset was used to determine the effect size and perform power calculations. Post-hoc analysis indicated that our initial experiment had a power of 78% to detect a significant difference between the groups. A priori power assessment indicates that a sample size of 6 and 9 per group is required to achieve 80% and 95% power respectively. We subsequently reproduced the initial dataset in a second experiment. This analysis confirmed our initial observation and effect size (p = 0.029 for calculated EEV (n = 4 vs 6), 0.0002 for Ashcroft score (n = 4 vs 6) and 0.0142 for hydroxyproline measurement (n = 4 vs 5) (Figure S2).

The agreement of the calculated EEV in the pulmonary fibrosis model with standard measurements was demonstrated by linear regression. Calculated EEV agreed with both the Ashcroft score (R^2^  = 0.523; p = 0.0002) (Figure 2E) and the hydroxyproline content (R^2^  = 0.598; p = 0.0004) (Figure 2F).

### Serial in vivo µCT imaging allows dynamic evaluation of fibrosis

**Figure 3:**
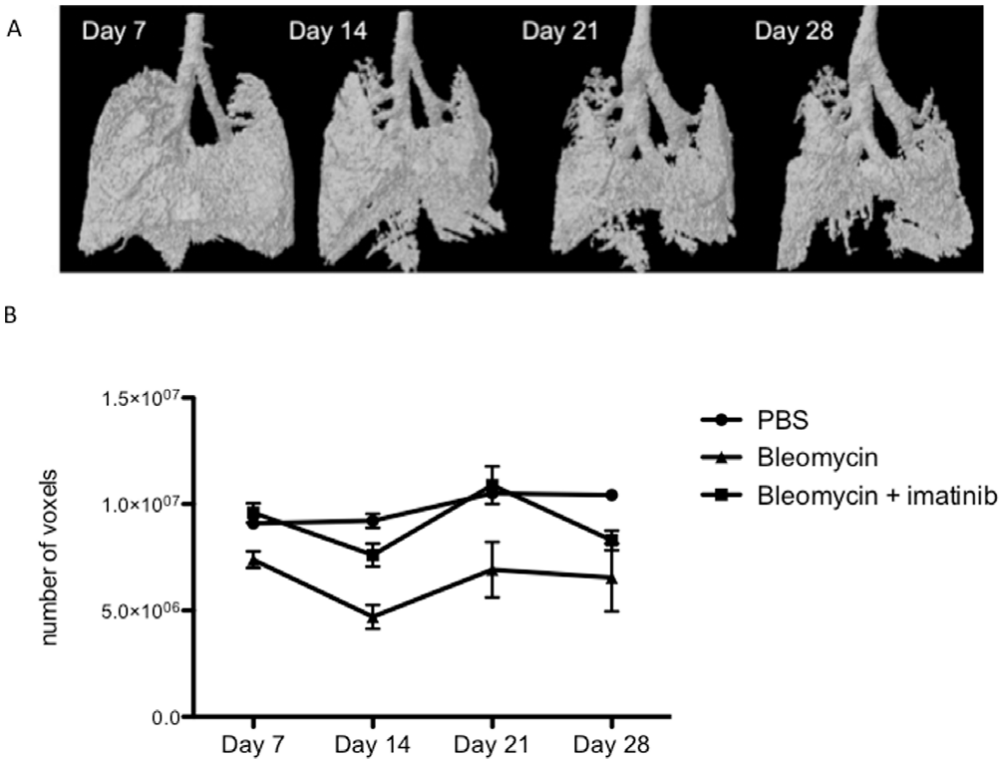
Serial µCT imaging during bleomycin-induced lung fibrosis. (A) 3D micro-CT imaging of progressive pulmonary fibrosis in a representative bleomycin-treated mouse over time. (B) Mean EEV over time after induction of fibrosis. (data are mean +/− SEM, differences were significant over time (p = 0.0061) and per group (p = 0.0291) when comparing treated vs non-treated mice exposed to bleomycin (n = 4 vs 5). Adding the non-bleomycin treated control group (n = 7) also showed significant interaction between time and treatment group (p = 0.007).

As compared to end-stage evaluations, serial *in vivo* imaging allowed longitudinal qualitative and quantitative follow-up of individual animals. Mice were scanned weekly, from day 7 to 28 after induction. Analysis of the longitudinal volume dataset generated extra topographical data on the dynamics and the extent of bleomycin-induced pulmonary fibrosis, with progressive, heterogeneous fibrosis visualized from day 7 onwards (Figure 3A).

In addition to descriptive images, changes in lung volumes over time may be useful to evaluate the effects of treatments or the impact of genetic models. Relatively small differences at the end-stage of the model limit the power of end-stage analysis. An anticipated 30% better preserved lung volume after imatinib treatment in the bleomycin model analyzed with t-test requires a sample size of 36 animals (19 per group) to achieve 80% power. A priori power analysis for 2-way ANOVA including time-repeated measurements with an estimated 30% reduction after imatinib intervention, indicates that a sample size of 4 per group is sufficient to achieve 80% power between repeated measurements. We used 2-way ANOVA to detect dynamic effects of imatinib in a small cohort of mice after fibrosis induction. EEV steadily increased over time in controls, reflecting normal growth; however a gradual decrease was seen in the bleomycin-treated mice, compatible with progressive restrictive pulmonary disease appearing from day 7 onwards, peaking 21 days after induction (Figure 3B). Effects were significantly different over time (p = 0.0061) and per group (imatinib vs. vehicle (n  = 4 vs 5) (p = 0.0291). Adding the non-bleomycin treated control group also showed significant interaction between time and treatment group (p = 0.007) (n = 7). Although the numbers of animals available in this experiment are limited and the experiment was slightly underpowered, these data suggest that longitudinal analysis may provide added value in screening approaches.

### µCT based radiologic pressure-volume (PV) relations are consistent with pulmonary function tests

**Figure 4:**
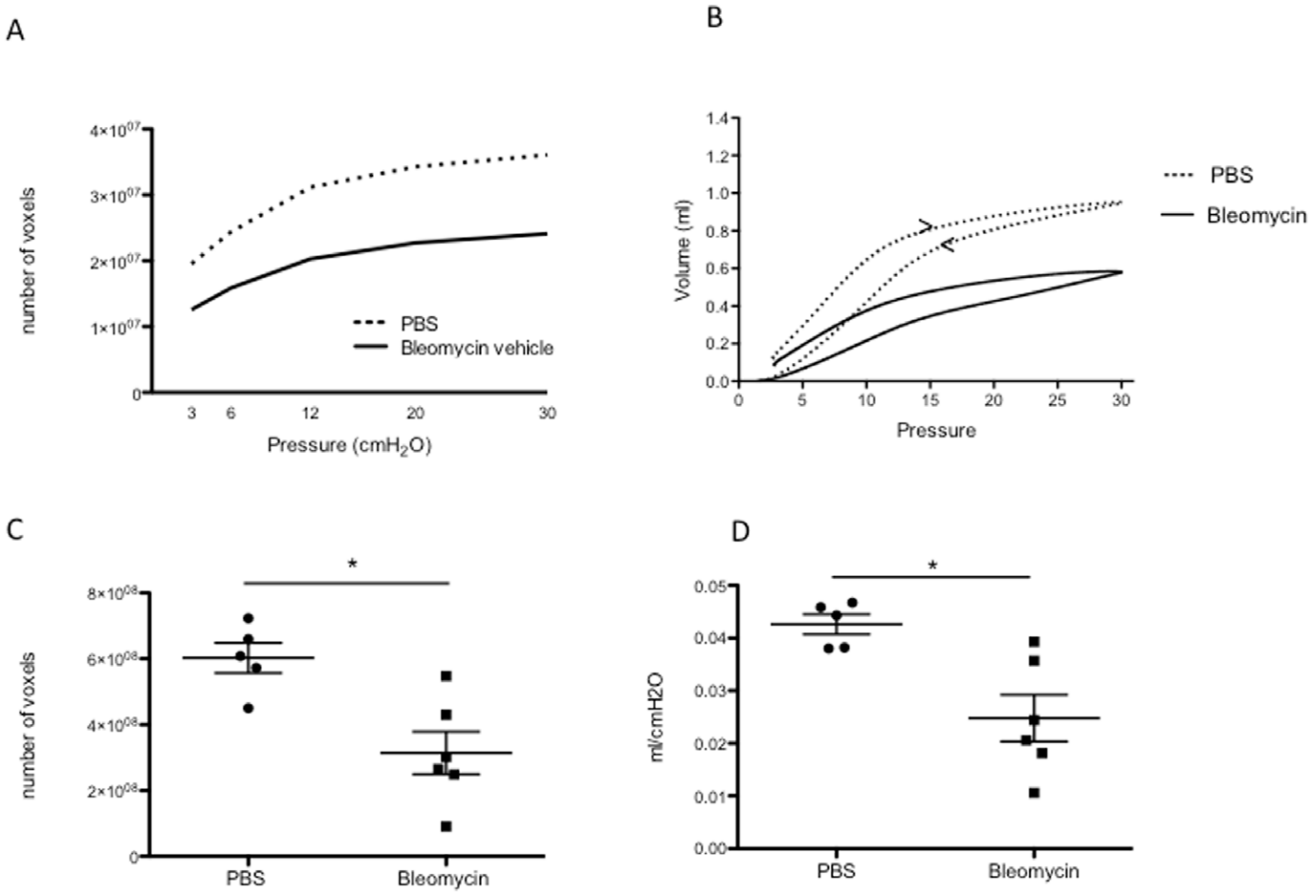
Pressure-Volume (PV) relationships. (A) PV-curves of bleomycin-induced pulmonary fibrosis, based on µCT data. (B) PV-loops in bleomycin-induced pulmonary fibrosis, based on flexiVent measurement, with inflating and deflating (<) curve. (C) Area under the curve (AUC) of µCT based PV-curves in pulmonary fibrosis (data are mean & 95% CI, *p = 0.0065 versus PBS). (D) Compliance in bleomycin-induced lung fibrosis, measured by flexiVent (data are mean & 95% CI, *p = 0.0102 versus PBS).

µCT imaging also allows the post-sacrifice construction of pressure/volume or voxel curves based on the application of decreasing pressures via the tracheal cannula and simultaneous scanning. The curve reconstructed from bleomycin-treated lungs shifted downward and flattened, corresponding to restrictive disease compared to controls (Figure 4A). The shape of the µCT image-derived PV curves and the deflating curve of the PV loops constructed with the flexiVent measurements were similar (Figure 4B). Area under the curve calculations allowed for quantification of the curves highlighting the significant effect of bleomycin (p  = 0.0065 – n = 5 vs 6) (Figure 4C). Compliance as measured in the snapshot perturbation with the flexiVent set-up, further corroborated our findings with µCT, Ashcroft score and hydroxyproline content and was significantly (p = 0.0102 – n = 5 vs 6) decreased in the bleomycin-treated animals (Figure 4D). Similar results were found for H (tissue elasticity) and G (tissue damping/resistance) as measured in the forced oscillation perturbation - P8 (data not shown).

### Further validation of µCT imaging in the elastase-induced model of lung emphysema

**Figure 5:**
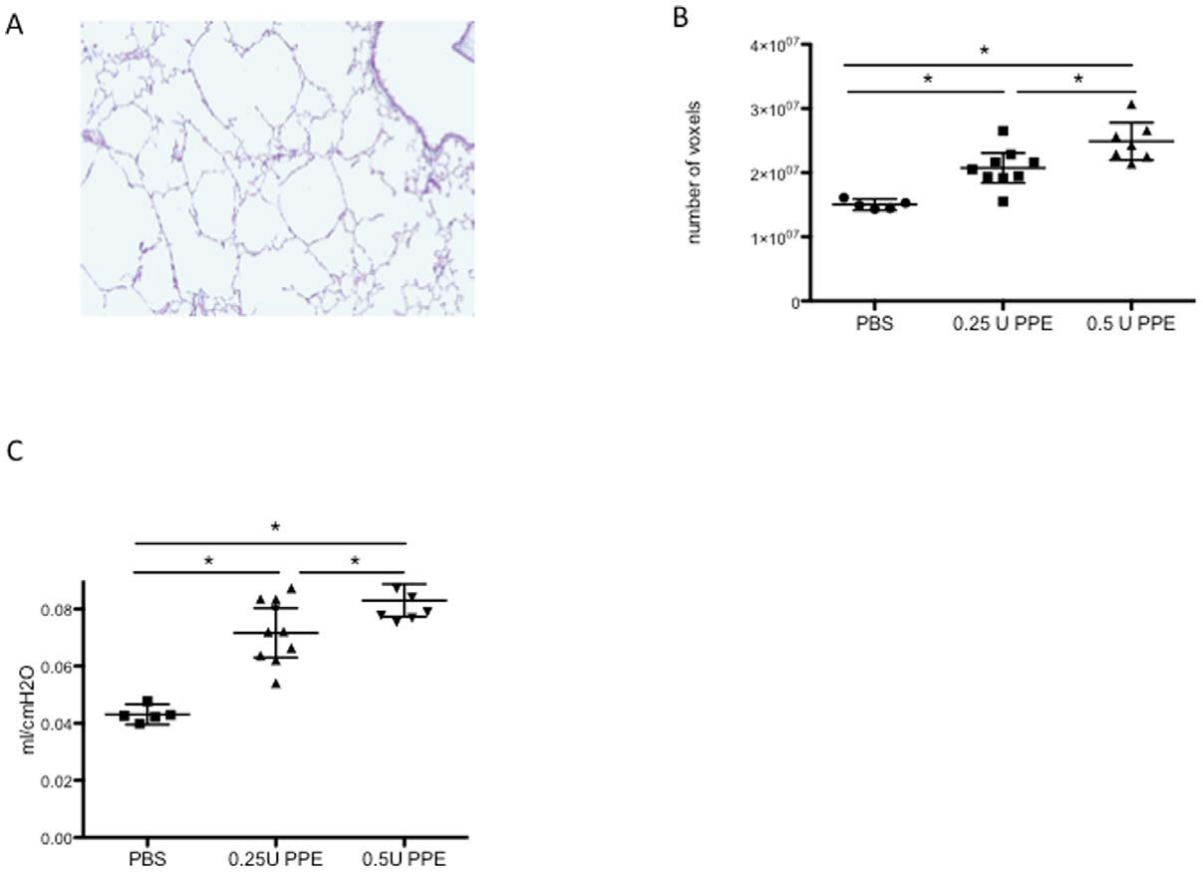
µCT imaging can also quantify lung emphysema in mice. (A) Representative histological image of an elastase-treated mouse (hematoxylin and eosin staining). (B) End-inspiratory aerated volumes (EEV), calculated by µCT, in elastase (PPE)-induced pulmonary emphysema (ANOVA p<0.0001; PBS versus both 0.25U and 0.5U PPE: * p<0.05, 0.25U versus 0.5U PPE: * p<0.05). (C) Pressure-Volume (PV)-curves of elastase-induced pulmonary emphysema, based on flexiVent measurement (ANOVA p<0.0001; PBS versus both 0.25U and 0.5U PPE: * p<0.05, 0.25U versus 0.5U PPE: * p<0.05).

Intra-tracheal instillation of elastase resulted in increased airway space as evidenced by histomorphological analysis (Figure 5A). In the elastase-treated mice, a dose-dependent increase in aerated lung volumes was observed based on µCT imaging quantification (1 way ANOVA p<0.0001 with all pair-wise post-hoc tests p<0.05 – n = 5,9 and 8 per group) (Figure 5B). These data were in line with compliance measurements using flexiVent pulmonary function tests. A dose-dependent increase in pulmonary compliance was observed after elastase treatment (1 way ANOVA p<0.0001 with all pair-wise post-hoc tests p<0.05 – n = 5,9 and 8 per group) (Figure 5C).

## Discussion

*In vivo* imaging in small animal models of human disease is a powerful tool to visualize disease processes, to assess their topographical distribution and to qualitatively evaluate severity in a non-invasive manner. Increases in resolution and quality of *in vivo* small animal CT technology have opened new perspectives for longitudinal quantification of pathological processes in preclinical research provided high-quality images and validated quantification can be established [9].

We validated a non-invasive, dynamic *in vivo* µCT analysis protocol for lung imaging and the evaluation of lung fibrosis and extended our approach to emphysema. Our protocol ran fully automated and resulted in quantification of aerated lung volume. These volumes were calculated based on stacked density histograms and showed good agreement with gold standard histopathology scores, total collagen content, and with physiological lung measurements obtained by invasive pulmonary function tests. We demonstrated that this imaging technique and consecutive automated analysis are suitable for the longitudinal and quantitative follow-up of dynamic changes in fibrotic and emphysematous pulmonary pathology.

*In vivo* pulmonary imaging in small animals is technically challenging due to movement artifacts caused by respiration. The image quality can be significantly improved by respiratory gating, either via a prospective or retrospective approach. Prospective respiratory gating implies triggering of the µCT apparatus to acquire images, at set phases in the respiratory cycle, resulting in images acquired in identical phase of the respiratory cycle, with minimal movement artifacts [9]. This can be achieved through intubation and connection to a mechanical ventilator. This approach has limitations, as it requires specific technical skills. Moreover, anesthesia and paralysis alter respiratory mechanics and repetitive intubation caries significant risks in mice [18]. A promising alternative is the use of a pressure transducer pad that detects diaphragm motions and generates pressure alterations in an air chamber under the abdomen of the mouse and regulate image acquisition [19]. Retrospective respiratory gating in contrast involves random image acquisition throughout the respiratory cycle, with post-acquisition sorting of the images into different groups, corresponding to a single respiratory phase. The respiratory cycle is reconstructed based on intrinsic or extrinsic gating techniques. Intrinsic, image-based gating protocols analyze the acquired images and generate surrogate signals for respiration [20]. Extrinsic retrospective respiratory gating implies detection of the respiratory movement, via pneumatic pillows or video graphic techniques. The degree of movement artifacts is largely reduced, albeit to a lesser extent as compared to the prospective approach. We show that retrospective gating results in high quality images that can be processed for further quantitative analysis.

Images allow intuitive visual assessment, comparable to what is common in clinical practice. This approach is valid and correlates with histopathological scoring, but requires specific training, is operator-dependent, semi-quantitative and remains time-consuming, as each image needs to be scored [6], [11]. To overcome these issues in rodent models of pulmonary disease, earlier semi-automated and automated µCT image analysis protocols have been proposed. In fibrotic disease, restriction of lung volumes is a hallmark feature. Quantification of the aerated lung volume thus indirectly serves as an indicator of the extent of fibrosis. The challenge lies in correct segmentation of the air-filled lung spaces from intrapulmonary fibrotic consolidations and from surrounding extrapulmonary tissues (e.g. mediastinal structures, diaphragm) considering limited, but inevitable movement.

The quality of the correct lung segmentation depends on the image quality, reflected by the image resolution. Resulting image resolution in *in vivo* imaging techniques is always a trade-off between resolution, contrast, radiation dosage and time. Cavanaugh et al. [9] performed µCT imaging in bleomycin-exposed mice at 90 µm resolution. Image analysis was based on semi-automated density thresholding, requiring the investigator's visual determination of the lung peak on the histogram and thus relatively labor-intensive. Only a weak non-signficant correlation was found between µCT image-based lung volume and mean histological damage. A lack of significant alterations in lung volumes in bleomycin-exposed animals versus controls was problematic in earlier publications, likely related to lower image resolution (100 µm) and the less sensitive point-grid image analysis method for lung segmentation [8], [10]. A more recent publication by Rodt et al [6] successfully demonstrated the inherent quality of lung imaging µCT, with significant correlations between µCT-derived aerated lung volumes and the histopathology scores in adenoviral TGFβ-induced lung fibrosis in mice. The authors compared both visual assessment and a semi-automated quantification algorithm, the latter still requiring manual placing of the seeding points for the region growing algorithm, and thus potentially causing observer-induced assessment bias.

We developed a fully automated analysis protocol, a timesaving approach that effectively eliminates observer interference. Moreover, we confirmed its validity as a quantitative measurement tool, with low inherent measurement error, resulting in lung volume data that agree with current gold standard evaluation techniques of histopathology and collagen quantification. We also demonstrated that this non-invasive technique is feasible for use in longitudinal experiments with repeated scanning of individual mice. This longitudinal analysis with the µCT image acquisition and analysis protocol highlighted the potential of the technique to detect mild treatment effects.

**Figure 6:**
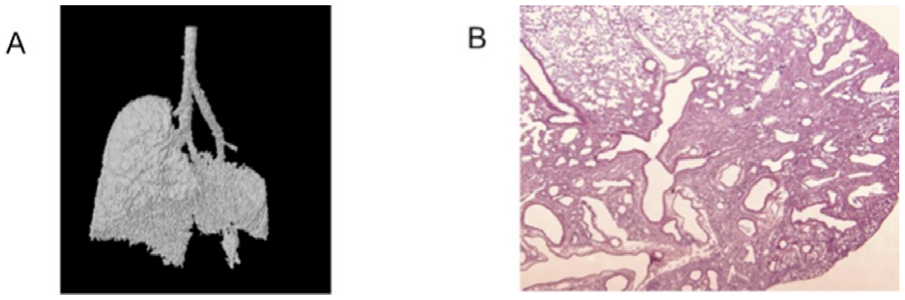
Specific topographic information conveyed by µCT imaging. (A) 3D image of an individual mouse identified as an outlier in the correlation between Ashcroft score and µCT derived end-inspiratory volume. The image shows an almost normal right lung, but selective damage to the upper part of the left lung, which was sampled for histology. (B) Corresponding histological image (hematoxylin and eosin staining) confirming disease in the upper part of the left lung.

Topographical information of pathological process can be very valuable. Distribution of emphysema in the elastase-induced model and of the fibrotic areas throughout the lungs in the bleomycin model is often heterogeneous, but µCT imaging can provide critical insights and furthermore explain conflicting results from an individual animal. The apparent paradox in a single mouse of a high Ashcroft score (scored on tissue sections from the left lung) with preserved µCT-derived aerated lung volume data, was visually elucidated by the 3D image depicting complete sparing of the right lung lobes and solitary involvement of the upper left lobe (Figure 6A), which was specifically studied in the histology analysis (Figure 6B) indicating that the induction of the model in this particular mouse was technically unsound leading to sample error.

Potential limitations to the proposed analysis include the availability of the technology, its validation across different µCT platforms and models, and the effects of repeated exposure to radiation. Micro-CT equipment is increasingly used in other scientific domains such as bone biology and disease [21], tissue engineering and material sciences [22], [23], suggesting that their availability for rodent imaging will become more widespread than pulmonary function instruments. Our algorithm was developed on a dedicated *in vivo* high-resolution scanner but the general setup is straightforward and could easily be adapted towards other commercially available instruments. We acknowledge that the bleomycin-induced model has many shortcomings as a preclinical model, since its pronounced inflammatory nature does not reflect the complete pathophysiology of human pulmonary fibrosis. Moreover, the short-term duration of the bleomycin model conflicts with the long-term, slowly progressive nature of human IPF. Here, we used this model solely as a paradigm for restrictive, fibrotic disease and as a tool to validate our µCT lung imaging technique.

As radiation-induced toxicity is a possible side effect, a subgroup of mice was serially scanned, without any other intervention. We detected no differences in these mice, compared to unscanned animals. Earlier reports suggested that a radiation dose of 13×0.62 Gy does not result in significant changes [24]. The radiation dose associated with our image acquisition protocol is lower (4×0.813 Gy), suggesting that the experimental conditions do not have direct toxic effects within the relatively short-time frame used in this study. One could object that we do not have baseline measurements. This was due, in part, to our wish not to increase the number of procedures, including anesthesia and radiation injury. Moreover we did include appropriate vehicle controls at each time point and the groups had similar body weights and hence similar lung volumes, at the time of induction.

To our knowledge, this is the first report of systematic, high-resolution longitudinal *in vivo* µCT imaging in the bleomycin-induced pulmonary fibrosis model in mice, showing its potential for quantitative 4D evaluation. We developed and validated a fully automated objective analysis protocol resulting in quantitative volume and density data that correlated to the current standard evaluation techniques. We confirmed the broader applicability and validity of the technique in the elastase-induced emphysema model. This approach opens new opportunities for the dynamic evaluation of pathological processes and putative therapeutic interventions within an individual animal.

## Supporting Information

Figure S1 **µCT imaging set-up.** (A) Image of the visual camera inside the CT apparatus, detecting up- en downward movement of the marker attached to the thorax. (B) Screenshot, depicting the input images from the camera, and the subsequently generated pseudo-sinusoidal curve reflecting breathing movements.
(TIFF)

Figure S2 **Confirmation of initial observations in a second data set.** (A) End-expiratory aerated volumes (EEV), calculated by µCT, in bleomycin-induced pulmonary fibrosis (data are mean & 95% CI, *p = 0.0299). (B) Ashcroft score (data are mean & 95% CI, *p = 0.0002). (C) Total collagen content (data are mean & 95% CI, *p = 0.0142).
(TIFF)
