# Supplementary figures and images for: Quantification of Lung Fibrosis and Emphysema in Mice Using Automated Micro-Computed Tomography
Source: PLoS One. 2012 Aug 13;7(8):e43123. doi: 10.1371/journal.pone.0043123 (PMC3418271; doi:10.1371/journal.pone.0043123)

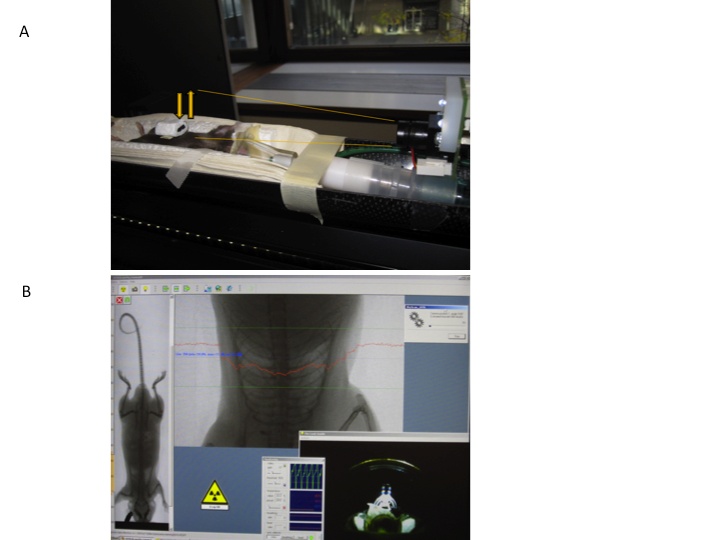

Supplement: Figure S1 — µCT imaging set-up. (A) Image of the visual camera inside the CT apparatus, detecting up- en downward movement of the marker attached to the thorax. (B) Screenshot, depicting the input images from the camera, and the subsequently generated pseudo-sinusoidal curve reflecting breathing movements. (TIFF) [file pone.0043123.s001.tiff]

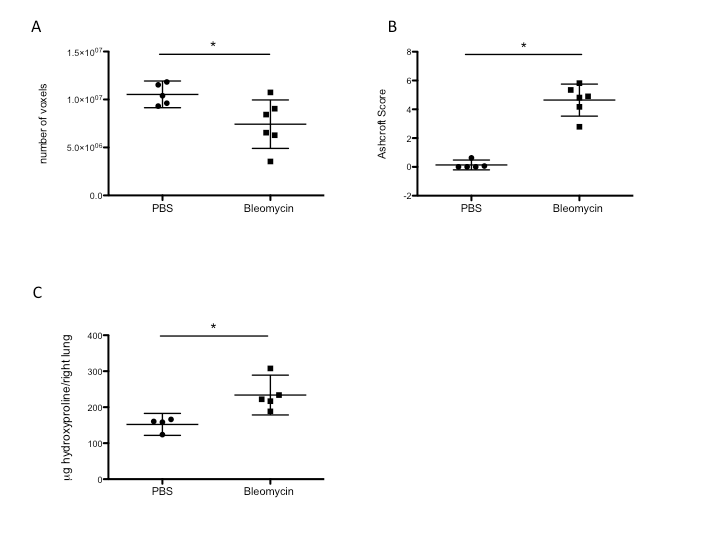

Supplement: Figure S2 — Confirmation of initial observations in a second data set. (A) End-expiratory aerated volumes (EEV), calculated by µCT, in bleomycin-induced pulmonary fibrosis (data are mean & 95% CI, *p = 0.0299). (B) Ashcroft score (data are mean & 95% CI, *p = 0.0002). (C) Total collagen content (data are mean & 95% CI, *p = 0.0142). (TIFF) [file pone.0043123.s002.tiff]
